# Supplementary material for: Identification of photoperiod-induced specific miRNAs in the adrenal glands of Sunite sheep (Ovis aries)
Source: Front Vet Sci. 2022 Jul 22;9:888207. doi: 10.3389/fvets.2022.888207 (PMC9354845; doi:10.3389/fvets.2022.888207)
Supplement: Supplementary file 1 [file Data_Sheet_1.ZIP › Supplementary file/miRDeep2 file.zip!/miRDeep2 file/Novel_Structure/SP42A2/Novel_470.pdf]

Provisional ID	:	NC_019484.2_43502
Score total	:	55.7
Score for star read(s)	:	3.9
Score for read counts	:	48.6
Score for mfe	:	1.7
Score for randfold	:	1.6
Score for cons. seed	:	
Total read count	:	107
Mature read count	:	103
Loop read count	:	0
Star read count	:	4

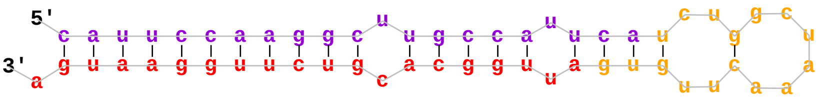

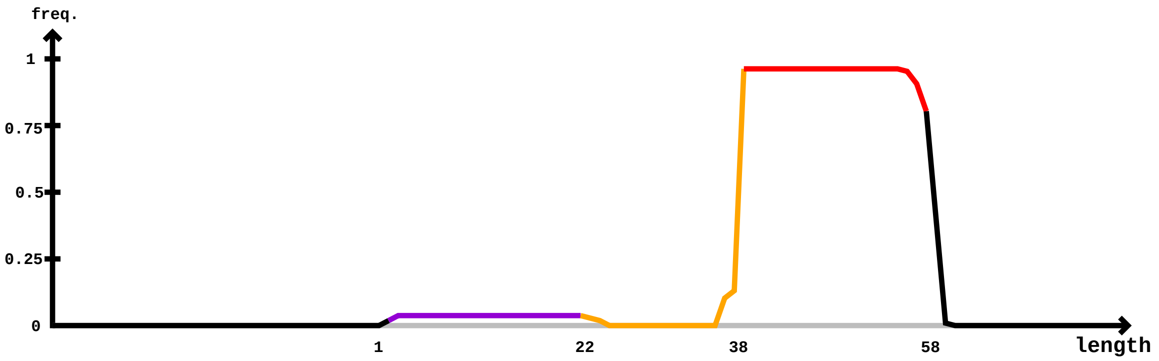

Star **Mature**

```
5'- uugccaccuucagcgugucugugugcuguaccccauuccaaggcuugccauucaucuggcuaaacuugugauuggcacgucuuggaaugaaguaacguacaacaaugaug  -3'   obs
    uugccaccuucagcgugucugugugcuguaccccauuccaaggcuugccauucaucuggcuaaacuugugauuggcacgucuuggaaugaaguaacguacaacaaugaug         exp
    ..((........))..((((((((((..(((..(((((((((((.(((((.((((..(......)..)))).))))).)))))))))))..)))..)))).)))..))).    reads     mm       sample
    .................................cauuccaaggcuugccauuca........................................................    2         0        seq
    ..................................auuccaaggcuugccauucauc......................................................    2         0        seq
    ....................................................................ugauuggcacgucuuggaa.......................    1         0        seq
    ....................................................................ugauuggcacgucuuggaau......................    3         0        seq
    ....................................................................ugauuggcacgucuuggaaug.....................    3         0        seq
    ....................................................................ugauuggcacgucuuggaauga....................    3         0        seq
    ....................................................................ugauuggcacgucuuggaaugaa...................    1         0        seq
    .....................................................................gauuggcacgucuuggaau......................    1         0        seq
    .....................................................................gauuggcacgucuuggaaug.....................    1         0        seq
    .....................................................................gauuggcacgucuuggaauga....................    1         0        seq
    ......................................................................auuggcacgucuuggaau......................    1         0        seq
    ......................................................................auuggcacgucuuggaaug.....................    7         0        seq
    ......................................................................auuggcacgucuuggaauga....................    59        0        seq
    ......................................................................auuggcacgucuuggaaugaa...................    21        0        seq
    ......................................................................auuggcacgucuuggaaugaag..................    1         0        seq
```
